# Risk factors and prediction model for new-onset hypertensive disorders of pregnancy: a retrospective cohort study

**DOI:** 10.3389/fcvm.2024.1272779

**Published:** 2024-05-01

**Authors:** Ling Zhou, Yunfan Tian, Zhenyang Su, Jin-Yu Sun, Wei Sun

**Affiliations:** ^1^ Department of Obstetrics and Gynecology, Liyang People's Hospital, Liyang, Jiangsu, China; ^2^ Department of Cardiology, The First Affiliated Hospital of Nanjing Medical University, Nanjing, China

**Keywords:** hypertensive disorders of pregnancy, body mass index, weight change, risk factors, prediction model

## Abstract

**Background and aims:**

Hypertensive disorders of pregnancy (HDP) is a significant cause of maternal and neonatal mortality. This study aims to identify risk factors for new-onset HDP and to develop a prediction model for assessing the risk of new-onset hypertension during pregnancy.

**Methods:**

We included 446 pregnant women without baseline hypertension from Liyang People's Hospital at the first inspection, and they were followed up until delivery. We collected maternal clinical parameters and biomarkers between 16th and 20th weeks of gestation. Logistic regression was used to determine the effect of the risk factors on HDP. For model development, a backward selection algorithm was applied to choose pertinent biomarkers, and predictive models were created based on multiple machine learning methods (generalised linear model, multivariate adaptive regression splines, random forest, and k-nearest neighbours). Model performance was evaluated using the area under the curve.

**Results:**

Out of the 446 participants, 153 developed new-onset HDP. The HDP group exhibited significantly higher baseline body mass index (BMI), weight change, baseline systolic/diastolic blood pressure, and platelet counts than the control group. The increase in baseline BMI, weight change, and baseline systolic and diastolic blood pressure significantly elevated the risk of HDP, with odds ratios and 95% confidence intervals of 1.10 (1.03–1.17), 1.10 (1.05–1.16), 1.04 (1.01–1.08), and 1.10 (1.05–1.14) respectively. Restricted cubic spline showed a linear dose-dependent association of baseline BMI and weight change with the risk of HDP. The random forest-based prediction model showed robust performance with the area under the curve of 0.85 in the training set.

**Conclusion:**

This study establishes a prediction model to evaluate the risk of new-onset HDP, which might facilitate the early diagnosis and management of HDP.

## 1 Introduction

Hypertensive disorders of pregnancy (HDP) is a common placental-mediated syndrome characterised by elevated blood pressure, proteinuria and edema (1). HDP can result in various serious complications, such as hemolysis, placental abruption, and stillbirth (2). Besides the short-term effects on pregnancy, HDP also increases the long-term risk of subsequent chronic hypertension and other cardiovascular diseases (3–5). Currently, HDP still constitutes a significant health issue globally due to the complexity of the conditions, the diversity of clinical presentations, and the lack of comprehensive prediction tools that facilitate early diagnosis and management (6). HDP is an idiopathic disease comprising gestational hypertension, preeclampsia, eclampsia, and pregnancy complicated by chronic hypertension. Chronic hypertension and gestational hypertension are the major components of HDP (7). Despite advancements in obstetrics and perinatal care, HDP continue to be a leading cause of maternal and neonatal mortality (1). The reported prevalence rates of HDP, gestational hypertension, and preeclampsia are 5.2%–8.2%, 1.8%–4.4%, and 0.2%–9.2% in all pregnancies, respectively (8). In the United States, HDP affects approximately one in nine pregnancies (9). Importantly, there has been a consistent rise in the incidence of gestational hypertension.

Compared with normotensive pregnancies, HDP resulted in an excess 202,400 hospital days and inpatient care costs of $366 million per year in the United States (9). The International Society for the Study of Hypertension in Pregnancy (ISSHP) has emphasized the importance of screening for gestational hypertension since gestational hypertension has the potential to progress to preeclampsia in the later stages of pregnancy (10). In light of the substantial health and financial burden, there has been a continuous effort to prevent and manage HDP (11). Previous studies have identified several risk factors for HDP, such as maternal age, multiple pregnancies, genetics, etc. However, none of these has been universally accepted as the definitive standard for HDP screening or prediction due to their insufficient discriminatory accuracy when considered individually (12). Also, recent prediction models have primarily focused on assessing the risk of preeclampsia but ignored gestational hypertension or new-onset hypertension (13, 14). Therefore, this study aims to identify the risk factors for new-onset HDP and to establish a prediction model to evaluate the risk of new-onset hypertension during pregnancy.

## 2 Methods

### 2.1 Participants' inclusion and exclusion

Adult pregnant women who underwent physical examinations and delivered at Liyang People's Hospital were continuously recruited for the study. The exclusion criteria included: (1) absence of baseline or follow-up blood pressure assessments; (2) pre-existing hypertension (chronic hypertension concurrent with pregnancy); (3) secondary hypertension; (4) severe cardiac, liver, or kidney dysfunction; and (5) autoimmune diseases. Ultimately, 446 participants without pre-existing hypertension were enrolled in the study. These participants were initially registered during their first prenatal visit (approximately at the 12th week of pregnancy) and were then monitored every four weeks until delivery.

### 2.2 Data collection

Maternal clinical parameters and serum biomarkers were collected after an overnight fast between the 16th and 20th weeks of gestation. These clinical parameters included age, height, baseline weight, weight change, baseline systolic blood pressure (SBP), baseline diastolic blood pressure (DBP), gravidity, and parity. Weight change was calculated as the difference between baseline weight and weight measured during the final examination. The body mass index (BMI) was calculated by weight(kg)/[height(m)]^2. SBP and DBP were measured thrice using an automatic blood pressure monitor following at least 30 min of rest, with the mean blood pressure being utilised for subsequent analyses. The fasting serum biomarkers included haemoglobin, leukocyte, and platelet counts.

### 2.3 The diagnosis of HDP

According to the 2018 ISSHP guideline, HDP is categorised into two types: (1) hypertension known before pregnancy or present in the first 20 weeks of gestation, and (2) hypertension arising *de novo* at or after 20 weeks (10). Following the 2018 ISSHP guidelines, our study defined new-onset hypertension as hypertension arising *de novo* at or after 20 weeks, encompassing both gestational hypertension and preeclampsia. The SBP ≥ 140 mmHg or DBP ≥ 90 mmHg was set a cut-off for hypertension. Participants who developed HDP were classified as the disease group, while those who did not develop HDP were designated as the control group.

### 2.4 Statistical analysis

The statistical analysis was conducted following the guidelines of the Scientific Publication Committee of the American Heart Association (15). The multivariate multiple imputation method was performed to fill in missing variates, which could minimise selection bias and improve statistical efficiency (16, 17). The "Multivariate Imputation by Chained Equations" package was applied and the default parameters were applied. Continuous variables adhering to a normal distribution (determined by the Kolmogorov-Smirnov test) were articulated as the mean ± standard deviation, whereas those with a skewed distribution were presented as a median along with the interquartile range. The representation of categorical variables was done through frequencies paired with percentages. For the comparison between the HDP group and the control group, we applied one-way ANOVA test (for normally distributed variables), the Kruskal-Wallis test (for skewed distribution), or chi-square test (for categorical variables) as appreciated.

Logistic regression was employed to evaluate the impact of the maternal clinical parameters and serum biomarkers on the development of HDP. Risk factors that achieved statistical significance (*P* < 0.05) in univariate logistic regression analyses were subsequently included in a multivariate logistic regression model. The effect sizes were presented using odd ratios (ORs) with 95% confidence intervals (CIs). Moreover, we illustrated the influence of waist circumference on new-onset hypertension via a restricted cubic spline (RCS) with 4 knots located at the 5th, 35th, 65th, and 95th percentiles. The 65th knots were set as the reference unless otherwise stated.

Moreover, to establish the prediction model for new-onset hypertension, we first screened the risk factors using an automatic backwards selection algorithm based on the Classification and Regression Training (caret) package (version 6.0–94) in R (18). Then, the identified risk factors were used to establish the prediction model using multiple machine learning methods, including the generalised linear model, multivariate adaptive regression splines, random forest, and k-nearest neighbours. The model performance was evaluated using the area under the curve. All participants were randomly allocated to either a training set or an internal validation set in an 8:2 ratio. The training set facilitated the selection of features and the training of the prediction model, while the internal validation set was employed to evaluate the model's performance. This cross-validation strategy ensures that the model is both trained and tested on independent subsets of the dataset, enhancing the generalizability and robustness of the predictive model.

A *P*-value < 0.05 was considered as statistical significance. All statistical analyses were conducted using R software (version 4.3.0).

## 3 Results

### 3.1 Characteristics of the study population

Table 1 summarises the characteristics of the participants in the HDP and control groups. Of the 446 pregnant participants without baseline hypertension, 153 developed new-onset HDP before delivery. We observed significant disparities in several baseline and physiological attributes, mainly favouring the development of HDP. Baseline weight, BMI, weight change during pregnancy, baseline SBP, and baseline DBP values were notably higher in the HDP group. Regarding pregnancy history, the HDP group contained a greater proportion of first-time pregnancies (44.4%) and participants without previous childbirth (60.1%) compared to the control group.

**Table 1:** Participants’ characteristics in the HDP group and control group.

	HDP group (*n* = 153)	Control group (*n* = 293)	*P*
Age (years)	29.0 (28.0, 30.0)	30.0 (29.0, 31.0)	0.501
Height (cm)	160.0 (160.0, 163.0)	160.5 (160.0, 162.0)	0.416
Baseline weight (Kg)	63.0 (60.0, 67.0)	60.0 (58.0, 61.0)	**<0** **.** **001**
Baseline BMI (Kg/m^2^)	24.2 (23.5, 25.7)	22.7 (22.0, 23.3)	**<0**.**001**
Weight change (Kg)	14.5 (14.0, 15.5)	12.0 (12.0, 13.0)	**0**.**006**
Baseline SBP (mmHg)	120.0 (118.0, 120.0)	110.0 (108.0, 110.0)	**<0**.**001**
Baseline DBP (mmHg)	79.0 (76.0, 80.0)	70.0 (70.0, 70.0)	**<0**.**001**
Gravidity:			**0**.**003**
1 time	44.4%	28.2%	** **
2 times	24.8%	28.2%	** **
Above 2 times	30.7%	43.5%	** **
Parity:			**0**.**001**
0 time	60.1%	41.2%	** **
1 time	34.0%	48.5%	** **
Above 1 time	5.9%	10.3%	** **
Hemoglobin (g/L)	129.0 (127.0, 133.0)	123.0 (122.0, 125.0)	**<0**.**001**
Leukocyte (10^9^/L)	8.5 (8.0, 9.1)	9.0 (8.6, 9.3)	**0**.**010**
Platelets (10^9^/L)	223.0 (212.0, 231.0)	199.5 (192.0, 208.0)	**<0**.**001**

HDP, Hypertensive disorders of pregnancy.

The bold values means statistical significance.

Moreover, haematological parameters also showed a significant difference between the two groups. The HDP group exhibited increased haemoglobin and platelet levels, while leukocyte counts were marginally lower than those in the control group. These observations indicate an intricate relationship between HDP and specific haematological parameters, adding another dimension to understanding HDP risk factors. However, the study found no significant differences in age and height between the HDP and control groups.

### 3.2 The association between risk factors and the new-onset HDP

Table 2 outlines the relationship between various risk factors and the incidence of new-onset HDP, as analysed by univariate and multivariate logistic regression. In univariate analysis, baseline weight (OR = 1.04, 95% CI: 1.02–1.06, *P* < 0.001), baseline BMI (OR = 1.13, 95% CI: 1.07–1.19, *P* < 0.001), weight change (OR = 1.05, 95% CI: 1.02–1.09, *P* = 0.006), baseline SBP (OR = 1.09, 95% CI: 1.06–1.11, *P* < 0.001), baseline DBP (OR = 1.13, 95% CI: 1.10–1.17, *P* < 0.001), hemoglobin (OR = 0.89, 95% CI: 0.81–0.97, *P* = 0.009), leukocyte (OR = 1.04, 95% CI: 1.02–1.06, *P* < 0.001), and platelets (OR = 1.01, 95% CI: 1.00–1.01, *P* < 0.001) were found to have a significant effect on the onset of HDP.

**Table 2:** Univariate and multivariate logistic regression on the risk factor of HDP.

	Univariate		Multivariate	
	OR (95% CI)	*P*	OR (95% CI)	*P*
Age (years)	0.99 (0.95, 1.03)	0.692		
Height (cm)	0.99 (0.95, 1.02)	0.445		
Baseline weight (Kg)	1.04 (1.02, 1.06)	<0.001		
Baseline BMI (Kg/m^2^)	1.13 (1.07, 1.19)	<0.001	1.10 (1.03, 1.17)	0.007
Weight change (Kg)	1.05 (1.02, 1.09)	0.006	1.10 (1.05, 1.16)	< 0.001
Baseline SBP (mmHg)	1.09 (1.06, 1.11)	<0.001	1.04 (1.01, 1.08)	0.008
Baseline DBP (mmHg)	1.13 (1.10, 1.17)	<0.001	1.10 (1.05, 1.14)	< 0.001
Gravidity				
1 time	Reference		Reference	
2 time	0.56 (0.33, 0.93)	0.026	1.13 (0.52, 2.45)	0.76
Above 2 times	0.45 (0.28, 0.72)	0.001	1.03 (0.4, 2.62)	0.96
Parity				
0 time	Reference		Reference	
1 time	0.48 (0.31, 0.74)	0.001	0.45 (0.2, 1.01)	0.051
Above 1 time	0.40 (0.17, 0.86)	0.019	0.74 (0.21, 2.55)	0.64
Hemoglobin (g/L)	0.89 (0.81, 0.97)	0.009	0.80 (0.71, 0.9)	< 0.001
Leukocyte (10^9^/L)	1.04 (1.02, 1.06)	<0.001	1.02 (1, 1.04)	0.03
Platelets (10^9^/L)	1.01 (1.00, 1.01)	<0.001	1.01 (1, 1.01)	< 0.001

HDP, Hypertensive disorders of pregnancy; OR, odd ratio; CI, confidence interval.

**Figure 1:**
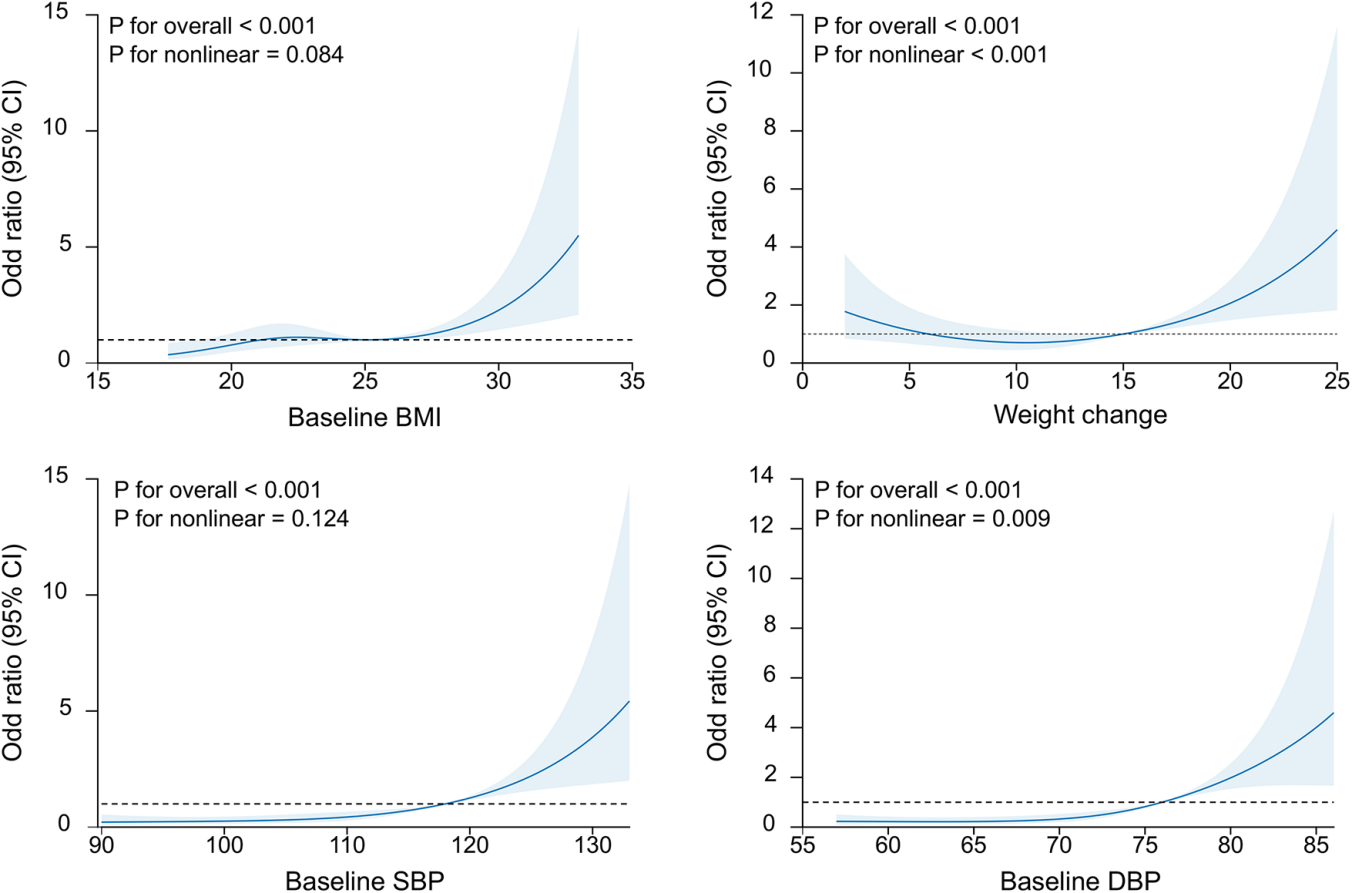
The restricted cubic splines of the association of baseline BMI, weight change, SBP and DBP with the new-onset HDP. BMI, body mass index; SBP, baseline systolic blood pressure; DBP, diastolic blood pressure.

Next, we input the risk factors with a *P* of <0.05 into the multivariate regression model (Table 2). In multivariate analysis, baseline BMI (OR = 1.10, 95% CI: 1.03–1.17, *P* = 0.007), weight change (OR = 1.10, 95% CI: 1.05–1.16, *P* < 0.001), baseline SBP (OR = 1.04, 95% CI: 1.01–1.08, *P* = 0.008), baseline DBP (OR = 1.10, 95% CI: 1.05–1.14, *P* < 0.001), hemoglobin (OR = 0.80, 95% CI: 0.71–0.9, *P* < 0.001), leukocyte (OR = 1.02, 95% CI: 1–1.04, *P* = 0.03), and platelets (OR = 1.01, 95% CI: 1–1.01, *P* < 0.001) remained significantly correlated to the risk of HDP. The baseline weight was not input into the multivariate analysis since the BMI was calculated based on body weight. However, other factors such as age, height, and number of pregnancies did not show any significant association. Furthermore, we illustrated the dose-dependent association between maternal clinical parameters and HDP risk (Figure 1). The RCS plots showed the significant effect of baseline BMI, weight change, baseline SBP, and baseline DBP on HDP (all *P* < 0.001). The linear dose-dependent relationship was observed in baseline BMI and baseline hypertension, whereas the U-shape relationship was observed between weight change and HDP and the J-shape curve was observed in baseline DBP. These results underscore the importance of monitoring physiological parameters such as BMI, weight change, baseline blood pressure, and blood cell count during pregnancy as potential predictive factors for new-onset HDP.

### 3.3 Construction and validation of the prediction model

The backwards selection algorithm of the caret package identified several features, including age, weight change, baseline SBP, baseline DBP, gravidity, parity, haemoglobin, leukocyte, and platelets. We established four distinct predictive models based on different machine learning methods (generalised linear model, multivariate adaptive regression splines, random forest, and k-nearest neighbours). In the training set, the area under the curve (AUCs) for the generalised linear model, multivariate adaptive regression splines, random forest, and k-nearest neighbours models were 0.87 (0.83–0.91), 0.91 (0.87–0.95), 0,99 (0.98–0.99), and 0.88 (0.84–0.92), respectively.

**Figure 2:**
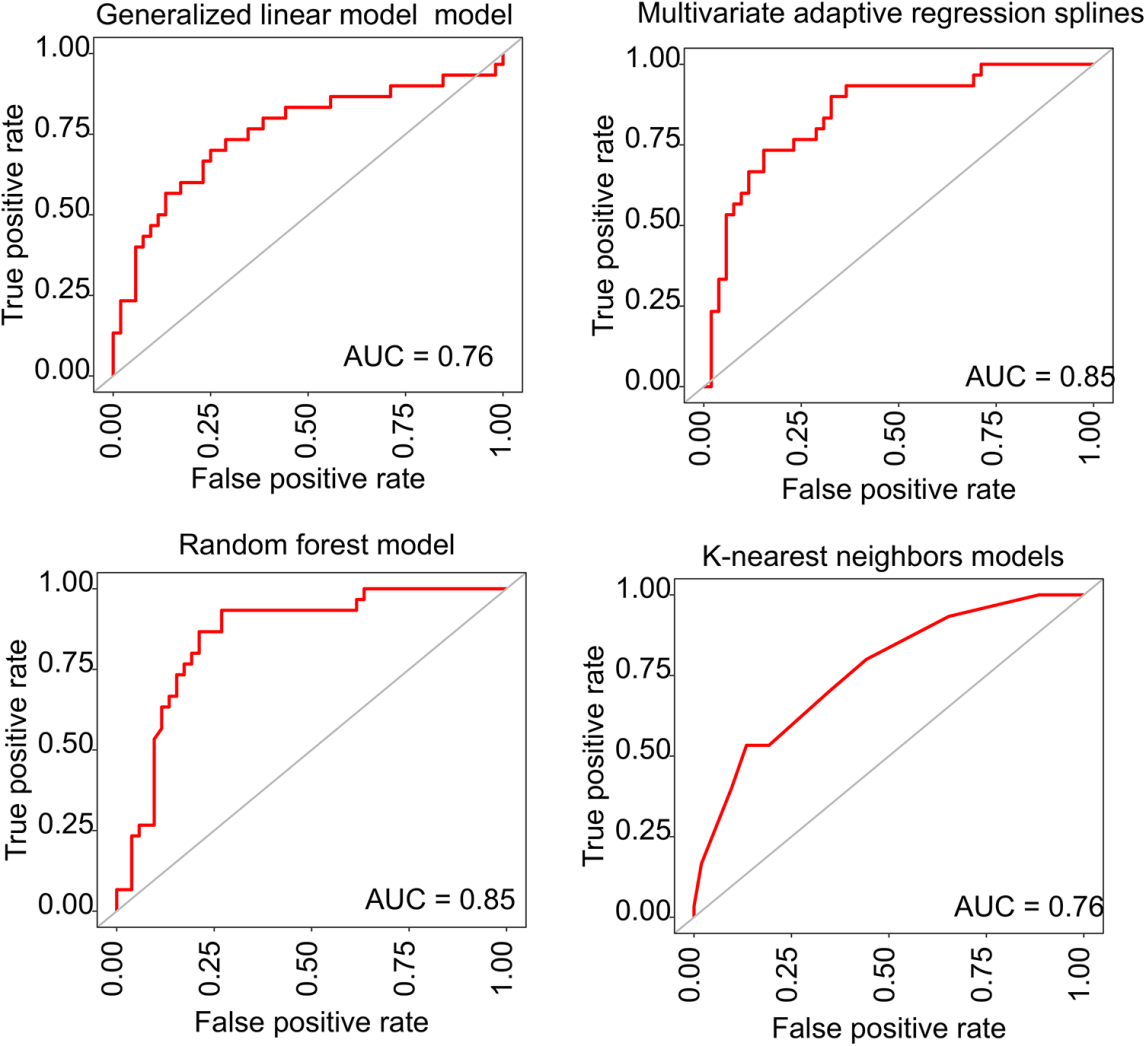
The ROC curves for the predictive performance of different prediction models. ROC, receiver operating characteristic; AUC, area under the curve.

In the internal validation set, the AUCs for the generalised linear model, multivariate adaptive regression splines, random forest, and k-nearest neighbours models were 0.76 (0.65–0.87), 0.85 (0.76–0.94), 0.85 (0.76–0.94), and 0.76 (0.65–0.87), respectively. The ROC curves of the proposed four models are given in Figure 2. The prediction models based on multivariate adaptive regression splines and random forest showed high performance with the area under the curve above 0.8 in the training set.

## 4 Discussion

Our study enrolled 446 participants free from baseline hypertension, of whom 153 developed new-onset HDP before delivery. Logistic regression analysis identified several risk factors for new-onset HDP, including baseline BMI, weight changes during pregnancy, baseline SBP and DBP, gravidity, parity, as well as haemoglobin, leukocyte, and platelet levels.

The escalating obesity rates have significantly amplified research interest in exploring the impact of baseline BMI and pregnancy-related weight changes as crucial risk factors. Particularly, maternal obesity has been identified as adversely affecting both maternal and neonatal outcomes. A comprehensive systematic review of 22 studies demonstrated that, compared to healthy controls, pregnant women with obesity face a markedly increased risk of pre-eclampsia, gestational hypertension, gestational diabetes, and depression (19). In the United States, obesity has become a leading contributor to the increasing incidence of pre-eclampsia over the last three decades (20). Similar findings were echoed in cohort studies from Canada (21) and Scotland (22), which highlighted a positive correlation between obesity and a heightened risk of HDP. Our study corroborates these observations, revealing a linear dose-response relationship between baseline BMI and the incidence of new-onset hypertension. Specifically, for every 1 kg/m^2^ increase in BMI, there is a corresponding 1.1-fold increase in the risk of developing HDP. Moreover, our analysis indicated no significant correlation between age and the onset of HDP, aligning with prior research (23, 24). However, this finding contrasts with some other studies. Paré et al. (25) revealed the advanced maternal age (above 40 years) as a significant risk factor for HDP development. Similarly, higher instances of pre-eclampsia and eclampsia have been reported in women over the age of 35 (26). The debate over maternal age as a risk factor for new-onset HDP suggests that the relationship remains unclear, underscoring the need for additional research to elucidate this association.

Although numerous epidemiological studies have focused on identifying risk factors associated with HDP, none of these biomarkers has been established as the definitive standard for HDP screening or prediction due to their limited discriminatory accuracy (12). Over the past decade, research on HDP focused on preeclampsia risk prediction but ignored gestational hypertension. Direkvand-Moghadam et al. (13) developed a preeclampsia prediction model based on factors such as previous preeclampsia, chronic hypertension, and infertility. This model reported an AUC of 0.67 (95% CI, 0.59–0.67). Poon and colleges (14) also established a prediction model assessing HDP risk based on pregnancy-associated plasma protein-A, placental growth factor, uterine artery pulsatility index and other parameters. Their model can predict early preeclampsia, late preeclampsia, and gestational hypertension with AUCs of 93.1%, 35.7%, and 18.3%, respectively.

To develop a predictive model for new-onset HDP, we employed a backward selection algorithm to screen features and subsequently created four distinct models using various machine learning methods. These models exhibited good performance in the internal validation set, with AUCs of 0.85 for both the multivariate adaptive regression splines model and the random forest model. Beyond their high efficacy and accuracy, the clinical features required in our model are readily assessable and cost-effective. Consequently, our prediction model holds promise for screening populations at high risk of new-onset HDP, facilitating timely access to disease management and interventions, thereby potentially enhancing maternal and neonatal outcomes. Still, it should be noted that the lack of external validation is a major limitation of this study, stemming from the unavailability of an external dataset. This absence hinders our capacity to evaluate the model's generalizability across varied populations and clinical settings, as all data were derived from a singular center. This limitation highlights the critical need for extensive validation to ascertain the relevance and applicability of our findings within diverse contexts. Future research should be conducted with a enlarged sample size to improve the predictive accuracy. Engaging in multicenter collaborations will be pivotal in enhancing the model's applicability and ensuring its utility in a range of clinical environments.

## 5 Conclusion

This study develops prediction models to evaluate the risk of new-onset HDP, potentially aiding in early diagnosis and management. The random forest-based prediction model demonstrated robust performance with an AUC of 0.85 in the training set. However, ongoing efforts are necessary to enhance predictive accuracy and to conduct additional external validations.

## Data Availability

The data used to support the findings of this study are available from the corresponding author upon request.
